# GPR182 and the reframing of lacteal chylomicron uptake

**DOI:** 10.1172/JCI207115

**Published:** 2026-06-15

**Authors:** Liqing Yu

**Affiliations:** Division of Endocrinology, Diabetes, and Nutrition, Department of Medicine, University of Maryland School of Medicine, Baltimore, Maryland, USA.

## Abstract

Historically, research on chylomicron entry into intestinal lymphatic vessels (lacteals) has been polarized between paracellular and transcellular transport models. In this issue of the *JCI*, Sun et al. identified GPR182 as a lipoprotein receptor in lymphatic endothelial cells (LECs), profoundly advancing our understanding of intestinal lipid absorption. They observed poor fat absorption in GPR182-deficient mice and demonstrated the role of GPR182 in transporting chylomicrons across the LECs into the lacteal lumen. This discovery establishes a molecular basis for transcellular transport of chylomicrons, challenging the traditional view that lacteal lipid entry is predominantly paracellular. By linking receptor-mediated uptake to impaired fat absorption and protection against fat-induced obesity and steatosis, this study expands the biological and translational implications of lacteal transport. Consequently, rather than favoring a single model, future research should investigate the integration of both paracellular and transcellular transport models in vivo.

## Chylomicron transport and the classical debate

Chylomicrons are large lipoproteins characterized by a hydrophobic core composed of triglycerides (85%–95%) and cholesterol esters, enveloped by a monolayer of phospholipids, free cholesterol, and structural proteins, notably apolipoprotein B-48. These lipoproteins are synthesized postprandially in absorptive enterocytes. Their biogenesis is crucial for the transport of dietary fats and fat-soluble vitamins into the circulation. Chylomicrons exit the small intestine by being secreted basolaterally from enterocytes into the interstitial space (lamina propria) and subsequently entering the intestinal lymphatic vessels (lacteals). From the lacteals, chylomicrons progress through the lymphatic system to the thoracic duct, and ultimately drain into the venous blood at the junction of the left subclavian and internal jugular veins. The mechanism by which dietary lipids cross the intestinal lacteal has remained a central question in gastrointestinal and vascular biology. For decades, the scientific community has debated two competing paradigms for chylomicron transport: a classical paracellular model, characterized by chylomicron passage through transient junctional openings between lymphatic endothelial cells (LECs), and a transcellular model, which proposes the internalization and transport of lipoproteins across the endothelial cell (EC) body. Notably, both models are substantiated by anatomical, histological, and physiological evidence (1–12).

## GPR182 as a transcellular receptor

GPR182 is an atypical chemokine receptor (ACKR5) characterized by constitutive association with β2-arrestin (13–15) and a broad chemokine binding profile (16). In this issue of the *JCI*, Sun et al. identified GPR182 as a critical lipoprotein receptor for efficient chylomicron uptake into lacteals by integrating GPR182-deficient mouse models, GPR182 blockade, and ultrastructural and cell-based mechanistic analyses (17). This study marks a pivotal advance by providing a molecular basis for a debate previously limited to morphological and physiological observations. Critically, the identification of GPR182 validates the plausibility of transcellular transport, necessitating a more nuanced reevaluation of the classical paracellular model. By establishing GPR182’s role in mediating chylomicron entry into the lacteals, the authors redefine the lacteal from a passive conduit to an active player of cargo recognition and transport. Ultimately, this conceptual shift offers a robust molecular framework for processes traditionally inferred largely through anatomical studies.

## Toward a dual-pathway model

The significance of the findings by Sun et al. is amplified when contextualized within the existing literature. A seminal review by Dixon on chylomicron uptake into lacteals underscored the unresolved nature of the transport mechanism, acknowledging the biological plausibility of both paracellular and transcellular pathways (7). Subsequently, Zhang et al. demonstrated that the “zippering” of lacteal junctions protects against diet-induced obesity, establishing that junctional architecture is a functional determinant of lipid absorption and metabolic outcomes (8). While the work of Zhang and colleagues reinforces the paracellular model, the characterization of *Gpr182*-KO mice by Sun et al. shifts the paradigm toward transcellular transport. Given that endothelial cells in other vascular beds are known to mediate macromolecule transport via receptor-dependent vesicular trafficking (18, 19), such a mechanism in lacteals is biologically credible. By identifying GPR182 as a critical molecular component, transcellular transport becomes experimentally tractable, prompting future investigations into its regulatory machinery and the specific physiological conditions under which it predominates.

**Figure 1:**
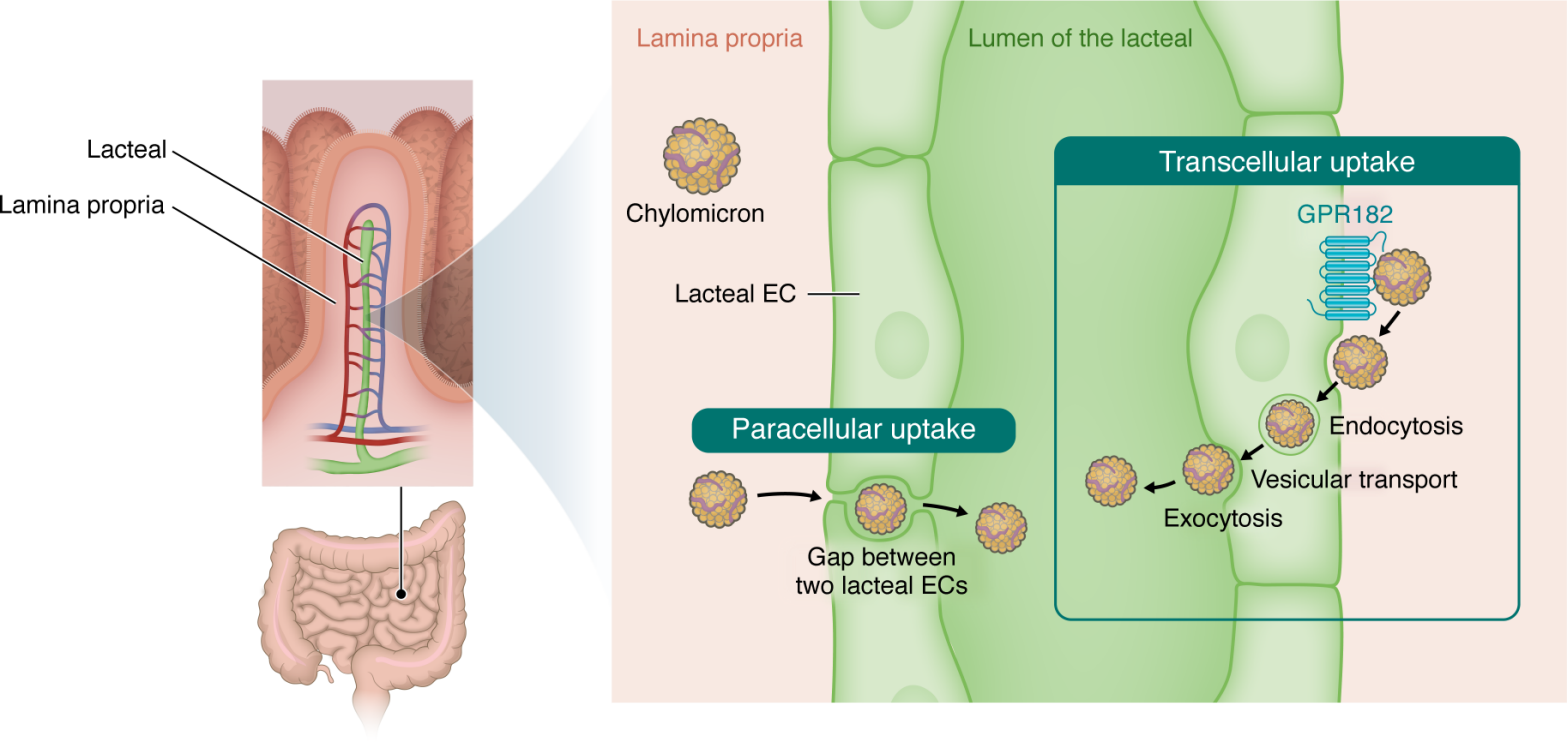
Paracellular and transcellular transport of chylomicrons into the lacteal. Chylomicrons in the lamina propria enter the lacteal lumen via two distinct routes. In the paracellular pathway, chylomicrons pass through the junctions between adjacent LECs. The findings of Sun et al. highlight the transcellular pathway’s contributions to chylomicron uptake, showing that chylomicrons bind to the GPR182 receptor, undergo endocytosis and vesicular transport through the LEC body, and are ultimately exocytosed into the lumen (17).

A particularly salient insight from recent literature is that classical paracellular and transcellular models should not be regarded as mutually exclusive. Zhang et al.’s study on lacteal zippering demonstrated that junctional tightening can significantly diminish chylomicron transport (8), while the GPR182 study suggests that receptor-mediated uptake represents a parallel mechanism (17). Together, these findings advocate for a layered model in which both structural permeability and vesicular transport are biologically relevant (Figure 1). This integrative perspective aligns more closely with the inherent complexity of lacteal biology than any single-route explanation.

## Redefining the lacteal

Beyond its therapeutic implications, the broader conceptual contribution of the study by Sun et al. is transformative. It necessitates a redefinition of the lacteal, transitioning from the view of this structure as a passive sieve to an active molecular interface. This shift is highly consequential, aligning the intestinal lymphatic vasculature with the contemporary understanding of endothelial cells as tissue-specific regulators of transport, signaling, and metabolism (20–23). The identification of GPR182 reinforces this principle by demonstrating that LECs utilize a dedicated receptor system for lipoprotein handling. Consequently, this study situates the lacteal within the broader framework of endothelial specialization that has revolutionized vascular biology in recent years.

## Translational potential

The translational implications of the findings by Sun et al. are equally profound. Intestinal lipid absorption is a fundamental determinant of systemic energy balance, postprandial lipemia, and susceptibility to metabolic disorders. Given that GPR182 may function as a rate-limiting factor in dietary lipid uptake, it represents a promising therapeutic target. The observation that GPR182 deficiency impairs lipid absorption, consequently protecting mice from diet-induced obesity and hepatic steatosis, suggests that this receptor regulates a pathway with broad systemic effects. This discovery introduces the prospect of modulating postprandial lipid flux at the lacteal level, offering a distinct mechanism from existing strategies that target appetite, enterocyte metabolism, or adipose energy storage. However, therapeutic inhibition requires careful consideration because lipid absorption is essential for nutrient homeostasis. Any intervention must preserve sufficient physiological function to avoid malabsorption, developmental complications, or fat-soluble vitamin deficiencies.

## Future directions

The next phase of research should prioritize integration rather than replacement. Several critical questions now arise. Is GPR182 necessary for all chylomicron uptake events or only a specific subset? Does its function fluctuate during fasting, during postprandial states, or under conditions of lipid overload? Furthermore, the specific endocytosis machinery it engages and its potential cooperation with caveolar, clathrin dependent, or other vesicular pathways require elucidation. It is also imperative to determine how GPR182 activity intersects with lacteal junctional remodeling and whether the relative contributions of paracellular and transcellular transport vary across intestinal regions, developmental stages, or disease states.

In the study by Sun et al., the elevation of blood HDL cholesterol (HDL-C) in GPR182-deficient mice is an intriguing observation (17). The mechanism underlying this phenomenon remains unknown. Intestinal ATP binding cassette transporter A1 (ABCA1) is known to contribute significantly to blood HDL-C levels (24). It remains to be determined whether chylomicron accumulation in the lamina propria induced by GPR182 deficiency leads to upregulation of the intestinal ABCA1 pathway. Additionally, given that global loss of the transcription factor pleomorphic adenoma gene like 2 (PlagL2) impairs lacteal uptake of chylomicrons (25), future studies should explore whether PlagL2 regulates paracellular or transcellular transport. Identifying a direct relationship between GPR182 and PlagL2 will determine whether GPR182 acts as a dominant transporter, a modulatory receptor, or one component of a broader transport program.

Future investigations should also examine whether lacteal transport is dynamically plastic rather than mechanistically fixed. Endothelial junctions and transcellular uptake pathways may be coordinated by dietary cues, inflammatory signals, metabolic hormones, and developmental programs. In this context, the lacteal may function less as a rigid conduit and more as a responsive metabolic gatekeeper.

## Conflict of interest

The author has declared that no conflict of interest exists.

## Funding support

This work is the result of NIH funding, in whole or in part, and is subject to the NIH Public Access Policy. Through acceptance of this federal funding, the NIH has been given a right to make the work publicly available in PubMed Central. This work was supported in part by the following:

- National Institute of Diabetes and Digestive and Kidney Diseases (NIDDK), NIH (DK111052 and DK116496, to LY).
- American Heart Association (17GRNT33670590, to LY).
- American Diabetes Association (award number 1-18-IBS-346, to LY).

## Version 1 06/15/2026

Electronic publication
